# Prognostic importance of mitosis quantification and PHH3 expression in oral epithelial dysplasia

**DOI:** 10.1007/s00428-023-03668-6

**Published:** 2023-10-26

**Authors:** Hrishikesh Sathyamoorthy, Hanya Mahmood, Amir Zaki Abdullah Zubir, Paul Hankinson, Syed Ali Khurram

**Affiliations:** 1 Unit of Oral and Maxillofacial Pathology, School of Clinical Dentistry, University of Sheffield, 19 Claremont Crescent, Sheffield, S10 2TA UK; 2 Academic Unit of Oral & Maxillofacial Surgery, School of Clinical Dentistry, University of Sheffield, 19 Claremont Crescent, Sheffield, S10 2TA UK

**Keywords:** PHH3; Phosphohistone-H3, Oral epithelial dysplasia, Mitosis, Malignant transformation, Recurrence, Oral squamous cell carcinoma

## Abstract

Oral epithelial dysplasia (OED) is diagnosed and graded using a range of histological features, making grading subjective and challenging. Mitotic counting and phosphohistone-H3 (PHH3) staining have been used for the prognostication of various malignancies; however, their importance in OED remains unexplored. This study conducts a quantitative analysis of mitotic activity in OED using both haematoxylin and eosin (H&E)-stained slides and immunohistochemical (IHC) staining for PHH3. Specifically, the diagnostic and prognostic importance of mitotic number, mitotic type and intra-epithelial location is evaluated. Whole slide images (WSI) of OED (*n* = 60) and non-dysplastic tissue (*n* = 8) were prepared for analysis. Five-year follow-up data was collected. The total number of mitosis (TNOM), mitosis type and intra-epithelial location was manually evaluated on H&E images and a digital mitotic count performed on PHH3-stained WSI. Statistical associations between these features and OED grade, malignant transformation and OED recurrence were determined. Mitosis count increased with grade severity (H&E: *p* < 0.005; IHC: *p* < 0.05), and grade-based differences were seen for mitosis type and location (*p* < 0.05). The ratio of normal-to-abnormal mitoses was higher in OED (1.61) than control (1.25) and reduced with grade severity. TNOM, type and location were better predictors when combined with histological grading, with the most prognostic models demonstrating an AUROC of 0.81 for transformation and 0.78 for recurrence, exceeding conventional grading. Mitosis quantification and PHH3 staining can be an adjunct to conventional H&E assessment and grading for the prediction of OED prognosis. Validation on larger multicentre cohorts is needed to establish these findings.

**Supplementary Information:**

The online version contains supplementary material available at 10.1007/s00428-023-03668-6.

## Introduction

Oral epithelial dysplasia (OED) describes a spectrum of histologically identified architectural and cytological disturbances involving the oral epithelium [1]. These lesions may progress to oral squamous cell carcinoma (OSCC) [2]. Higher grade lesions have higher risk of transformation, highlighting the need for an early and accurate diagnosis [1]. OSCC is the most common malignant neoplasm of the oral cavity associated with a myriad of environmental aetiologies and genetic alterations [3–5].

Because of the direct relationship between OED and malignant transformation, the dysplasia grade is considered the most important prognosticator for malignant transformation [5]. However, the current grading system (WHO, 2017) is associated with poor reproducibility, which can result in an inconsistent and unreliable diagnosis [6]. Suggestions to mitigate these shortcomings include the use of clinical determinants and molecular markers [7]. The binary grading system is an alternative criteria proposed to improve observer reproducibility by quantifying the minimum number of cytological and architectural features required for a diagnosis [8]. However, this classification uses the same histological features listed in the WHO Classification, and there remains a lack of high-quality evidence to support the prognostic importance of many of these features [2]. The recent update from the 5th Edition of the WHO Classification includes additional features, such as apoptotic mitoses and single cell keratinisation. However, the clinical relevance for inclusion of these features is unclear [9]. A recent study explored histological feature-specific associations in OED with clinical outcomes. The predictive performance of the proposed models for OED progression exceeded conventional grading [10]. However, a more detailed and prospective analysis of individual histological features is still needed to establish a more objective predictive/grading system.

**Fig. 1:**
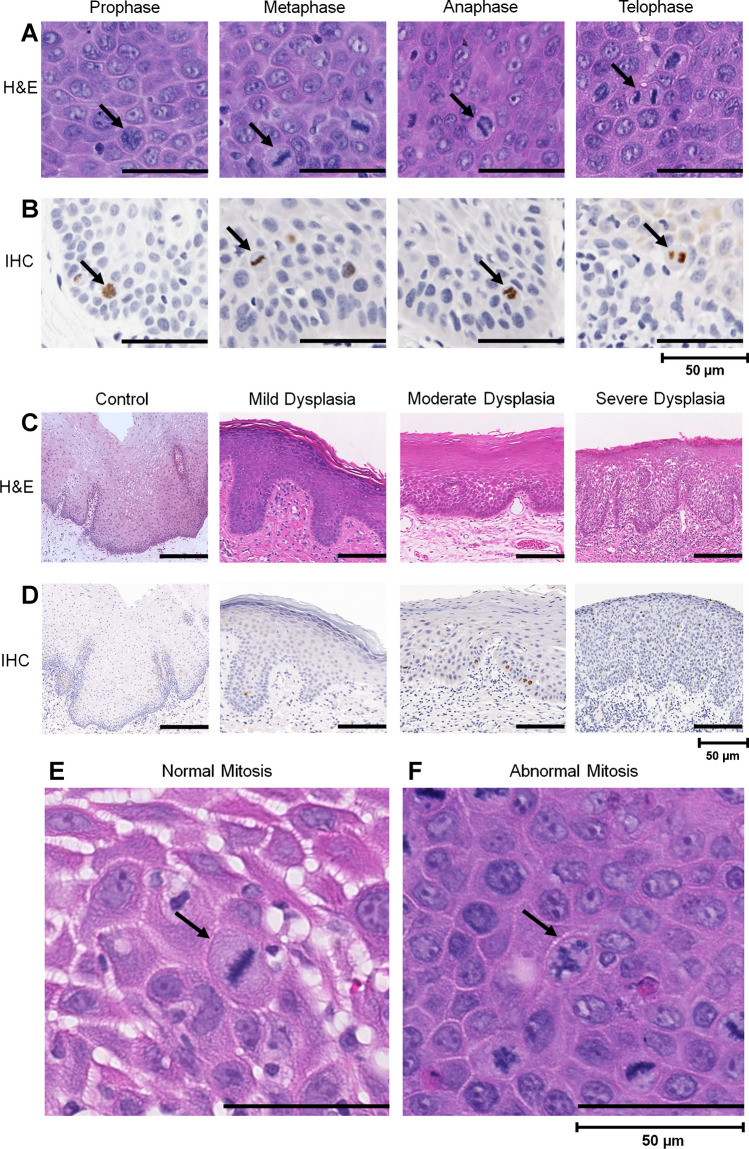
Photomicrographs (40 ×) demonstrating the different mitotic stages observed in OED (black arrows) based on H&E (**A**) and PHH3-IHC staining (**B**). Photomicrographs (20 ×) demonstrating the different OED grades (WHO, 2017) on H&E (**C**) and PHH3-IHC-stained images (**D**). H&E photomicrographs (40 ×) demonstrating ‘normal’ appearance of mitosis (**E**) and ‘abnormal’ appearance of mitosis (**F**) highlighted by black arrow

Mitotic figure counting is used for diagnosis and prognostication of various malignancies [11–14] including breast, gastric and neuroendocrine carcinomas [13, 15–17]. However, its importance in precancer diagnosis and progression is yet to be explored. The main limitation of mitosis counting is the tediousness of the manual approach, in addition to interpretation differences due to variations in chromatin arrangements in the different mitotic stages, and the resemblance of apoptotic bodies and pyknotic nuclei with mitotic bodies (Fig. 1) [18]. Many of these limitations can now be overcome by the increasing number of digital/computational tools which allow for automated quantification, providing more objective, efficient and reliable outputs [19]. However, in the case of mitotic cell counting, attention also needs to be given to the presence of abnormal mitotic forms, characterised by mitotic asymmetry or an abnormal segregation of chromosomes [20].

Various biomarkers have been implicated in OED progression, but the evidence to support their routine use is still lacking [21]. Phosphohistone-H3 (PHH3) is a specific protein phosphorylated during chromatin condensation in mitosis [22]. It stains positively during the late G2 phase and M phase. Phosphorylation of the histone H3 starts to occur just before prophase which is not identifiable on haematoxylin and eosin (H&E) examination [18], lending to the role of PHH3 a useful marker.

The aims of this study were threefold: first, to conduct a quantitative analysis of mitotic activity in OED (including number, type and intra-epithelial location of mitoses) using digitised H&E sections and immunohistochemical (IHC)-stained tissue with PHH3; second, to evaluate changes in mitotic activity relative to OED progression; and third, to develop and explore multivariable models using mitotic features for prediction of OED recurrence and malignant transformation, with comparison to conventional grading.

## Material and methods

### Case selection and tissue processing

Following ethical approval (reference 18/WM/0335), a retrospective sample of 68 H&E-stained tissue sections were retrieved from the department archive. The sample comprised OED sections (*n* = 60) of varying grades (mild, moderate, severe) with 5-year post-diagnosis data, in addition to non-dysplastic control samples (*n* = 8) which included cases of benign hyperplasia, scar tissue and inflammatory oral lichen planus. Verrucous and HPV-related OED lesions were excluded based on morphological features, as they are distinct entities with reportedly different behaviours.

Prior to the inclusion, cases were independently reviewed by a consultant oral and maxillofacial pathologist (SAK) to ensure there was sufficient epithelial tissue for analysis. Cases with insufficient tissue, gross artefact or tangentially cut sections were excluded. All cases were then blindly re-evaluated by SAK, HM (clinician with extensive expertise and specialist interest in OED analysis) and PH (trainee oral and maxillofacial pathologist) to confirm the original diagnosis and where necessary assign an updated OED grade (using WHO and binary systems). Grading variability was measured by a Cohen’s kappa score, which resulted in a value of 0.900, demonstrating good interobserver agreement.

New 5-μm-thick formalin-fixed paraffin-embedded sections of the selected cases were obtained for H&E and IHC staining. The sections were scanned at 40 × magnification using an Aperio-CS2 scanner (Leica Biosystems, Milton Keynes, UK) to obtain high-resolution whole slide images (WSI) producing 68 H&E slides and 67 IHC slides for analysis. The IHC sample had one less case due to technical scanning/imaging difficulties, resulting in its exclusion at the final stage.

Clinical data collection included patient age at diagnosis, sex, biopsy site, original histological grade (WHO, 2017), status of malignant transformation and recurrence (lesion that progressed to OSCC or recurred at the same clinical site following treatment within 5 years).

### Immunohistochemical staining for PHH3

IHC staining was carried out for the mitosis marker PHH3 (Ser10) using a previously described protocol [23]. A primary rabbit anti-human PHH3 polyclonal antibody (#9701; Cell Signalling Technology, 1:100 dilution) and a secondary goat anti-rabbit antibody was used. Following IHC, counterstaining with haematoxylin and mounting in DPX was done for further analysis.

### Analysis of mitosis activity in OED

QuPath software (v.0.3.2) was used for identification of regions of interest (ROI) and subsequent mitotic feature analysis [24]. For all slides, five rectangular-shaped ROIs of a consistent size (area≈165,000 mm^2^) corresponding to representative dysplastic and non-dysplastic regions were selected at 20 × magnification and verified by two experienced clinicians (HM, SAK).

For the H&E sample (*n* = 68), two observers (HS, SAK), blinded to clinical outcomes, were asked to independently count and record (i) the total number of mitoses (TNOM), (ii) the number of ‘normal’ and ‘abnormal’ mitoses and (iii) the intra-epithelial mitosis location (‘basal’ or ‘suprabasal’) in each field. An agreement between the observers was made on how to qualify a ‘normal’ and ‘abnormal’ mitosis. An equational bipartition of the chromosomal material was used as standard for ‘normal’ mitosis [25], whereas the presence of abnormalities like binucleation, pyknotic nuclei, micronuclei and broken-egg appearances qualified the mitoses to be ‘abnormal’ [26]. A kappa score of 0.646 was obtained between the two observers for independent mitosis counting. In cases of wide disagreement, a consensus score was agreed/used for the downstream analyses. The means and standard deviation for the mitosis variables (TNOM, type and location) from the five ROIs were recorded and an average obtained for each case.

For the PHH3-IHC sample (*n* = 67), QuPath’s inbuilt ‘positive cell detection’ algorithm was applied for automated quantification of positively stained mitoses, and intra-epithelial mitosis location recorded through manual assessment (by HS, SAK). Due to the nature of the automated detection, the mitosis type could not be confirmed in the IHC sample. All data were exported onto a pre-structured spreadsheet in Microsoft Excel**®** (v.2206).

### Statistical analyses

Statistical analyses were conducted in GraphPad Prism (v9) and IBM SPSS Statistics (v29.0.1.0). Data was tested for normality following which appropriate statistical tests were selected. Unpaired Student’s *t*-tests and one-way ANOVA were performed to compare differences in the TNOM, mitosis type and intra-epithelial location between OED grades and relative to control. Where relevant, an appropriate post hoc analysis (Tukey’s/Dunnett’s) was performed for pairwise comparisons. For the H&E analysis, the mean mitosis number and ratio of normal-to-abnormal mitoses were measured and compared between grades. Paired sample *t*-tests were conducted to compare the number of normal and abnormal mitoses across OED grades.

**Fig. 2:**
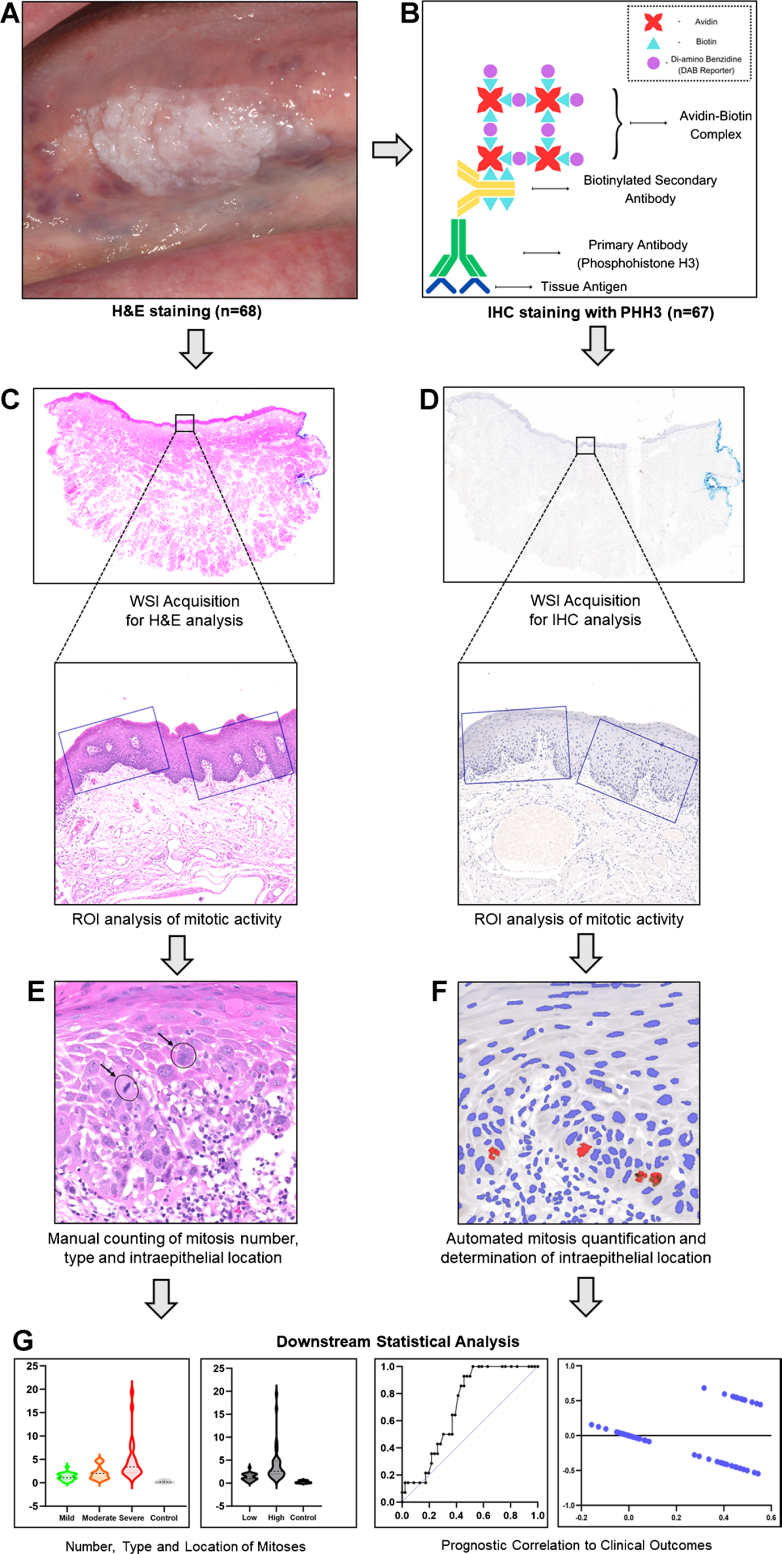
Overall workflow methodology of the study. **A** Identification, retrieval and preparation of H&E sample (*n* = 68). **B** Preparation of PHH3-IHC sample (*n* = 67). Conversion of tissue sections to digital WSI and identification of ROI for H&E (**C**) and PHH3-IHC analysis (**D**). **E** Manual assessment of mitosis activity (number, type, location) on H&E. **F** Automated mitosis quantification for PHH3-IHC sample. **G** Statistical analysis to assess mitotic activity in OED with correlation to clinical outcomes

Multivariable logistic regression models were explored separately for H&E and PHH3-IHC samples, to assess statistical relationships between individual and combined mitotic variables (TNOM, mitosis type, intra-epithelial location) with clinical outcomes (malignant transformation and OED recurrence). The effect of adding clinical variables (age, sex, intraoral site) and histological grade (WHO, binary) on model performance was assessed. The area under the receiver operator characteristic (ROC) curve was used to assess model accuracy and visualise performance. A *p* value of < 0.05 was considered statistically significant. Figure 2 depicts the workflow methodology for this study.

## Results

### Characteristics of the OED cohort

Amongst the 60 OED cases, 39 (65%) were male, and 21 (35%) were female, with a mean age of 61.73 years (IQR 18.5). The clinical intraoral site distribution was the tongue *n* = 28 (46.67%), floor of mouth *n* = 15 (25%), buccal mucosa *n* = 8 (13.33%), gingivae *n* = 5 (8.33%) and palate *n* = 4 (6.67%). The WHO histological grade distribution (following blind re-analysis) was mild OED = 20 (33.33%), moderate OED = 17 (28.33%) and severe OED = 23 (38.33%). Binary grade distribution was low-grade OED = 25 (41.7%) and high-grade OED = 35 (58.3%). A total of 14 cases (23.33%) transformed to OSCC, amongst which 8 (57.1%) were moderately dysplastic and 6 (42.9%) were severely dysplastic. Of the 19 (31.67%) cases that recurred after treatment, 8 were moderately dysplastic (42.1%), and 11 were severely dysplastic (57.9%).

### Analysis of H&E and PHH3 mitotic count

**Fig. 3:**
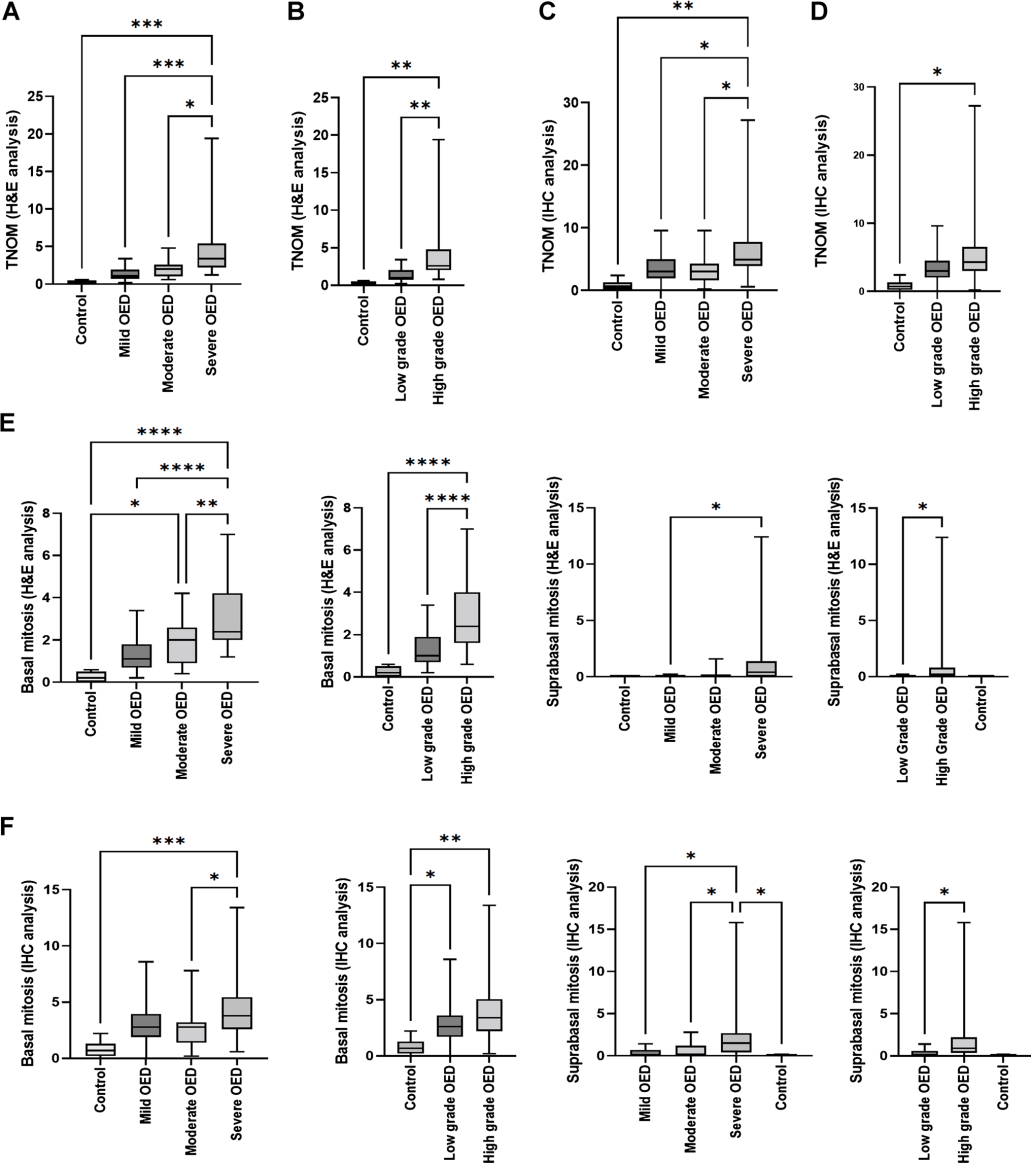
Analysis of the TNOM based on H&E sections (**A**,** B**) and PHH3-IHC sections (**C**,** D**) with comparisons between histological grades and relative to control. Analysis of intra-epithelial mitosis location based on H&E sections (**E**) and PHH3-IHC sections (**F**) with comparisons between histological grade and relative to control. Asterisk indicates a statistically significant finding (*p ≤ 0.05, **p ≤ 0.01, ***p ≤ 0.001, **** *p* ≤ 0.0001)

Both the H&E and IHC analyses yielded a statistically significant difference in the TNOM between WHO grades (H&E: *p* = 0.0005; IHC: *p* = 0.0073) and binary OED grades (H&E: *p* = 0.0012; IHC: *p* = 0.0403) (Fig. 3). A significant difference was also seen when comparing TNOM between the following groups: mild OED vs severe OED (H&E: *p* = 0.0006; IHC: *p* = 0.0197), moderate OED vs severe OED (H&E: *p* = 0.0113; IHC: *p* = 0.0181), severe OED vs control (H&E: *p* = 0.0004; IHC: *p* = 0.0009) and high-grade OED vs control (H&E: *p* = 0.0022; IHC: *p* = 0.0064) (Fig. 3).The remaining pairwise comparisons (mild OED vs moderate OED, mild OED vs control, moderate OED vs control and low-grade OED vs control) were not statistically significant. The mean mitosis number increased with grade severity (H&E: mild OED 1.32, moderate OED 2.09, severe OED 4.93, low-grade OED 1.32, high-grade OED 4.07) and relative to control (0.20). A similar trend was seen in IHC analysis (mild OED 3.47, moderate OED 3.26, severe OED 7.16, low-grade OED 3.36, high-grade OED 5.84, control 0.825).

### H&E analysis of mitosis type

#### Normal mitotic figures

There was a significant difference in the average number of ‘normal’ mitoses between WHO grades (*p* = 0.0016) and binary grades (*p* = 0.0040) (Table 1). Significant differences were also seen between the following groups: control vs severe OED (*p* = 0.0004), control vs high-grade OED (*p* = 0.0023), mild OED vs severe OED (*p* = 0.0026) and moderate OED vs severe OED (*p* = 0.0143) (Table 1).

**Table 1:** H&E analysis of mitosis type (measured by the presence and number of ‘normal’ and ‘abnormal’ mitoses) between individual grades of dysplasia (WHO and binary) and relative to control

Test parameters	Normal mitoses		Abnormal mitoses	
	*p* value	95% CI	*p* value	95% CI
Mild vs moderate OED	0.9051	− 1.655 to 1.158	0.5612	− 1.674 to 0.6705
Mild vs severe OED	0.0026*	− 3.198 to − 0.5906	0.0010*	− 2.811 to − 0.6383
Moderate vs severe OED	0.0143*	− 3.009 to − 0.2817	0.0322*	− 2.360 to − 0.08647
Control vs mild OED	0.3911	− 2.527 to 0.7569	0.9543	− 1.579 to 1.159
Control vs moderate OED	0.2359	− 2.817 to 0.5490	0.4349	− 2.114 to 0.6909
Control vs severe OED	0.0004*	− 4.390 to − 1.168	0.0032*	− 3.278 to − 0.5920
Control vs low-grade OED	0.3361	− 2.391 to 0.7048	0.8349	− 1.535 to 1.031
Control vs high-grade OED	0.0023*	− 3.774 to − 0.7875	0.0116*	− 2.806 to − 0.3312

Asterisk indicates statistical significance

#### Abnormal mitotic figures

Similar trends were seen for the presence of ‘abnormal’ mitoses between WHO grades (*p* = 0.0010) and binary grades (*p* = 0.0016) (Table 1) in addition to comparisons between control vs severe OED (*p* = 0.0032), control vs high-grade OED (*p* = 0.0116), mild OED vs severe OED (*p* = 0.0010) and moderate OED vs severe OED (*p* = 0.0322) (Table 1).

#### Normal-to-abnormal mitosis ratio

The ratio of normal-to-abnormal mitoses was higher in OED (1.61) compared to control (1.25). This ratio was found to reduce with increasing grade severity. The ratios for mild, moderate and severe grades were 3.26, 1.49 and 1.43, and for low and high grades, 2.75 and 1.44, respectively. Statistically significant differences were observed when comparing the ratio of normal/abnormal mitoses across different grades (*p* = 0.0001 mild OED, *p* = 0.0289 moderate OED, *p* = 0.0470 severe OED, *p* < 0.0001 low-grade OED, *p* = 0.0137 high-grade OED).

### Analysis of H&E and PHH3 mitosis location

#### Basal mitoses

A higher number of basal mitoses were observed with increasing grade severity, for WHO (mild OED = 1.3, moderate OED = 1.905882353, severe OED = 3.269565217) and binary grading (low-grade OED = 1.296, high-grade OED = 2.89) and relative to control (0.2) on H&E assessment (*p* < 0.0001). A similar trend was also seen on PHH3-IHC assessment between WHO grades and relative to control (*p* = 0.0287) (Fig. 3). Further comparisons demonstrated significance differences between mild OED vs. severe OED (H&E: *p* < 0.0001), moderate OED vs. severe OED (H&E: *p* = 0.0076; IHC: *p* = 0.0383), moderate OED vs. control (H&E: *p* = 0.0163), severe OED vs. control (H&E: *p* < 0.0001; IHC: *p* = 0.0005), low-grade OED vs. control (IHC: *p* = 0.0495) and high-grade OED vs. control (H&E: *p* < 0.0001; IHC: *p* = 0.0024) (Fig. 3). The remaining pairwise comparisons were not statistically significant.

#### Suprabasal mitoses

An increasing number of suprabasal mitoses were also observed with grade severity. Significant differences were shown between WHO grades (H&E: *p* = 0.0174; IHC: *p* = 0.0076) and binary grades (H&E: *p* = 0.0364; IHC: *p* = 0.0202) as well as between the following groups: mild OED vs. severe OED (H&E: *p* = 0.0302; IHC: *p* = 0.0123), moderate OED vs. severe OED (only IHC: *p* = 0.0446) and severe OED vs. control (only IHC: *p* = 0.0435) (Fig. 3). The remaining pairwise comparisons were not statistically significant.

### Multivariable model development exploration

The association between mitosis variables, clinical characteristics, histological grades and clinical outcomes was assessed (for H&E and PHH3-IHC analysis) using multiple logistic regression. For comparative purposes, the prognostic strength of conventional grading systems (WHO and binary) was also evaluated (Tables 2 and 3).

**Table 2:** Exploration of multivariate prognostic models based on the TNOM, mitosis location, clinical variables and histological grading systems (H&E *n* = 68, PHH3-IHC *n* = 67 − 5 ROI per WSI)

Model features	H&E models						PHH3-IHC models					
	Malignant transformation			OED recurrence			Malignant transformation			OED recurrence		
	AUROC	*p* value	95% CI	AUROC	*p* value	95% CI	AUROC	*p* value	95% CI	AUROC	*p* value	95% CI
*WHO grading*	0.6537	0.0836	0.5163 to 0.7911	0.7202	0.0064*	0.5950 to 0.8453	0.6635	0.0665	0.5266 to 0.8004	0.7316	0.0043*	0.6074 to 0.8557
*Binary grading*	0.6786	0.0444*	0.5289 to 0.8282	0.6893	0.0191*	0.5501 to 0.8286	0.6841	0.0388*	0.5347 to 0.8336	0.6961	0.0156*	0.5569 to 0.8352
TNOM	0.5753	0.3966	0.4197 to 0.7309	0.6297	0.1085	0.4798 to 0.7795	0.5468	0.5992	0.3837 to 0.7099	0.5197	0.8077	0.3606 to 0.6766
TNOM + WHO grading	0.7065	0.0201*	0.5766 to 0.8364	0.7401	0.0030*	0.6180 to 0.8621	0.746	0.0058*	0.6074 to 0.8847	**0.7783**	0.0006*	**0.6635 to 0.8930**
TNOM + binary grading	0.722	0.0124*	0.5817 to 0.8624	0.6926	0.0171*	0.5585 to 0.8266	0.7484	0.0053*	0.5992 to 0.8976	0.7184	0.0071*	0.5821 to 0.8548
TNOM + age	0.5776	0.3822	0.4203 to 0.7350	0.6412	0.0805	0.4989 to 0.7835	0.6063	0.2326	0.4346 to 0.7781	0.6276	0.1156	0.4820 to 0.7733
TNOM + sex	0.5233	0.7932	0.3643 to 0.6823	0.586	0.287	0.4327 to 0.7393	0.596	0.2811	0.4286 to 0.7634	0.5395	0.6265	0.3819 to 0.6971
TNOM + age + sex	0.6685	0.0579	0.4908 to 0.8462	0.629	0.1103	0.4767 to 0.7814	0.6587	0.0748	0.4728 to 0.8446	0.6395	0.0855	0.4850 to 0.7940
TNOM + clinical site	0.5652	0.4629	0.4089 to 0.7215	0.5757	0.3485	0.4305 to 0.7210	0.5786	0.3778	0.4212 to 0.7360	0.5237	0.7703	0.3678 to 0.6796
Basal mitoses	0.5815	0.3588	0.4256 to 0.7375	0.6175	0.1459	0.4674 to 0.7676	0.6381	0.1211	0.4744 to 0.8018	0.5411	0.5868	0.3825 to 0.7057
Basal mitoses + WHO grading	0.6793	0.0435*	0.5421 to 0.8166	0.7246	0.0054*	0.5997 to 0.8496	0.7643	0.003*	0.6141 to 0.945	0.7737	0.0007*	0.6540 to 0.8934
Basal mitoses + binary grading	0.7057	0.0206*	0.5607 to 0.8508	0.697	0.0147*	0.5638 to 0.8303	**0.7714**	0.0023*	**0.6260 to 0.9169**	0.7401	0.0031*	0.6111 to 0.8691
Basal mitoses + age	0.5885	0.3191	0.4286 to 0.7485	0.6483	0.0664	0.5058 to 0.7907	0.6627	0.0678	0.5008 to 0.8246	0.6362	0.0932	0.4915 to 0.7809
Basal mitoses + sex	0.5753	0.3966	0.4113 to 0.7393	0.6085	0.1793	0.4583 to 0.7587	0.6381	0.1211	0.4751 to 0.8011	0.5539	0.506	0.3928 to 0.7151
Basal mitoses + age + sex	0.6157	0.1929	0.4342 to 0.7972	0.6406	0.0818	0.4923 to 0.7888	0.681	0.0422*	0.5118 to 0.8501	0.6526	0.0599	0.5001 to 0.8051
Basal mitoses + clinical site	0.5854	0.3364	0.4297 to 0.7411	0.6085	0.1793	0.4632 to 0.7537	0.6254	0.1593	0.4610 to 0.7898	0.5401	0.6208	0.3804 to 0.6998
Suprabasal mitoses	0.5388	0.6622	0.3642 to 0.7135	0.5854	0.2906	0.4261 to 0.7446	0.6278	0.1515	0.4727 to 0.7828	0.6217	0.1335	0.4686 to 0.7748
Suprabasal mitoses + WHO grading	0.736	0.0079*	0.6130 to 0.8591	**0.7458**	0.0023*	**0.6249 to 0.8667**	0.6794	0.0441*	0.5462 to 0.8125	0.7533	0.0018*	0.6331 to 0.8735
Suprabasal mitoses + binary grading	**0.7562**	0.0039*	**0.6192 to 0.8932**	0.6945	0.0161*	0.5521 to 0.8369	0.6746	0.05	0.5124 to 0.8368	0.6816	0.0252*	0.5355 to 0.8276
Suprabasal mitoses + age	0.5877	0.3234	0.4285 to 0.7470	0.6168	0.1481	0.4734 to 0.7602	0.5532	0.5506	0.3862 to 0.7201	0.6625	0.0451*	0.5245 to 0.8005
Suprabasal mitoses + sex	0.5839	0.3453	0.4315 to 0.7362	0.5122	0.88	0.3611 to 0.6633	0.5103	0.9078	0.3427 to 0.6779	0.5414	0.6094	0.3872 to 0.6957
Suprabasal mitoses + age + sex	0.6584	0.0746	0.4846 to 0.8322	0.6175	0.1459	0.4639 to 0.7710	0.6389	0.119	0.4547 to 0.8231	0.6447	0.0744	0.4959 to 0.7936
Suprabasal mitoses + clinical site	0.5963	0.2785	0.4249 to 0.7676	0.5398	0.6223	0.3875 to 0.6920	0.5492	0.5807	0.3911 to 0.7073	0.5355	0.6614	0.3812 to 0.6898

The first two rows indicate the prognostic values for existing grading systems for comparative purposes. Highlighted rows indicate the most predictive models overall. Asterisk indicates a statistically significant finding. *AUROC* area under receiver operating characteristic

Text in bold indicate the most significant values/models

**Table 3:** Exploration of multivariate prognostic models based on the type of mitoses, clinical variables and histological grading systems on H&E assessment (*n* = 68 − 5 ROI per WSI)

Model features	H&E models					
	Malignant transformation			OED recurrence		
	AUROC	*p* value	95% CI	AUROC	*p* value	95% CI
*WHO grading*	*0.6537*	*0.0836*	*0.5163 to 0.7911*	*0.7202*	0.0064*	*0.5950 to 0.8453*
*Binary grading*	*0.6786*	0.0444*	*0.5289 to 0.8282*	*0.6893*	0.0191*	*0.5501 to 0.8286*
Normal mitoses	0.5016	0.9861	0.3383 to 0.6648	0.5552	0.4944	0.3983 to 0.7121
Normal mitoses + WHO grading	0.7469	0.0055*	0.6229 to 0.8709	0.7548	0.0016*	0.6358 to 0.8738
Normal mitoses + binary grading	0.7663	0.0027*	0.6479 to 0.8847	0.7298	0.0044*	0.6053 to 0.8543
Normal mitoses + age	0.5901	0.3107	0.4386 to 0.7415	0.6354	0.0936	0.4897 to 0.7812
Normal mitoses + sex	0.58	0.3681	0.4306 to 0.7294	0.5019	0.981	0.3478 to 0.6561
Normal mitoses + age + sex	0.6693	0.0568	0.4979 to 0.8406	0.6316	0.1033	0.4804 to 0.7827
Normal mitoses + clinical site	0.5613	0.49	0.4082 to 0.7145	0.552	0.5198	0.4041 to 0.6999
Abnormal mitoses	0.6856	0.0367*	0.5441 to 0.8270	0.7022	0.0123*	0.5648 to 0.8396
Abnormal mitoses + WHO grading	0.6537	0.0836	0.5169 to 0.7906	0.7163	0.0074*	0.5901 to 0.8425
Abnormal mitoses + binary grading	0.6475	0.0968	0.4777 to 0.8173	0.6444	0.0738	0.4942 to 0.7947
Abnormal mitoses + age	0.6071	0.2278	0.4532 to 0.7611	0.6515	0.0608	0.5139 to 0.7890
Abnormal mitoses + sex	0.632	0.1374	0.4760 to 0.7880	0.6354	0.0936	0.4874 to 0.7834
Abnormal mitoses + age + sex	0.6281	0.1493	0.4502 to 0.8060	0.6393	0.0847	0.4912 to 0.7874
Abnormal mitoses + clinical site	0.6328	0.1351	0.4802 to 0.7853	0.6207	0.1352	0.4809 to 0.7605
Abnormal mitoses + suprabasal mitoses	0.7616	0.0032*	0.6162 to 0.9071	0.6823	0.024*	0.5311 to 0.8335
Abnormal mitoses + suprabasal mitoses + WHO	**0.7888**	0.0012*	**0.6762 to 0.9014**	**0.7715**	0.0008*	**0.6554 to 0.8876**
Abnormal mitoses + suprabasal mitoses + TNOM	0.7803	0.0016*	0.6331 to 0.9275	0.6643	0.0419*	0.5114 to 0.8172
Abnormal mitoses + suprabasal mitoses + TNOM + WHO	**0.8113**	0.0005*	**0.6987 to 0.9239**	**0.7747**	0.0007*	**0.6598 to 0.8896**
Abnormal mitoses + basal mitoses	0.6157	0.1929	0.4636 to 0.7677	0.5796	0.3245	0.4234 to 0.7358
Abnormal mitoses + basal mitoses + WHO	0.6778	0.0454*	0.5407 to 0.8149	0.7208	0.0063*	0.5950 to 0.8466
Abnormal mitosis + basal mitoses + TNOM	0.7143	0.0159*	0.5500 to 0.8786	0.6457	0.0713	0.4893 to 0.8021
Abnormal mitosis + basal mitoses + TNOM + WHO	0.764	0.003*	0.6406 to 0.8873	**0.7895**	0.0003*	**0.6777 to 0.9013**

The first two rows indicate the prognostic values for existing grading systems for comparative purposes. Highlighted rows indicate the top most predictive models overall. Asterisk indicates a statistically significant finding. *AUROC* area under receiver operating characteristic

Text in bold indicate the most significant values/models

#### Prognostic potential of TNOM on H&E and PHH3-IHC sections

The TNOM alone had a modest association with malignant transformation (H&E: AUROC 0.5753; IHC: 0.5468) and OED recurrence (H&E: AUROC 0.6297; IHC: 0.5197), though the strength of association increased when combined with WHO grading (H&E: AUROC 0.7065 for transformation, 0.7401 for recurrence; IHC: AUROC 0.7460 for transformation, 0.7783 for recurrence) and binary grading (H&E: AUROC 0.722 for transformation, AUROC 0.6926 for recurrence; IHC: AUROC 0.7484 for transformation, AUROC 0.7184 for recurrence). The addition of clinical variables to TNOM had little or no effect on model performance (Table 2).

#### Prognostic potential of mitosis location on H&E and IHC-PHH3 sections

‘Basal’ mitosis was modestly associated with malignant transformation (H&E: AUROC 0.5815; IHC: AUROC 0.6381) and recurrence (H&E: AUROC 0.6175; IHC: 0.5411). In comparison, ‘suprabasal’ mitosis had a marginally weaker prognostic association (H&E: AUROC 0.5388 for transformation, AUROC 0.5854 for recurrence; IHC: 0.6278 for transformation, AUROC 0.6217 for recurrence). Whilst the addition of clinical variables had little overall effect on the prognostic strength of mitosis location, the incorporation of histological grading improved predictive strength, particularly for ‘suprabasal’ mitoses on H&E (‘suprabasal mitoses’ + ‘WHO grade’ = AUROC of 0.736 for transformation and 0.7458 for recurrence) (Table 2).

#### Prognostic potential of mitosis type on H&E sections

‘Abnormal’ mitoses alone had a greater predictive strength than ‘normal’ mitoses on H&E for transformation (AUROC 0.6856 vs 0.5016, respectively) and recurrence (AUROC 0.7022 vs 0.5552, respectively). However, incorporation of histological grading improved the predictive strength for ‘normal’ mitoses to a greater extent than for ‘abnormal’ mitoses (‘normal mitoses’ + ‘WHO grade’ = AUROC 0.7469, *p* = 0.0055 vs ‘abnormal mitoses’ + ‘WHO grade’ = AUROC 0.6537, *p* = 0.0836). The addition of clinical variables had little or no effect on model performance (Table 3).

#### Prognostic models using combined mitosis features

Combining the different mitosis variables with histological grading produced the most predictive models. The most superior model for prediction of transformation (‘abnormal mitoses’ + ‘suprabasal mitoses’ + ‘TNOM’ + ‘WHO grade’) produced an AUROC of 0.8113 (*p* = 0.0005, 95% CI 0.6987 to 0.9239), and the most superior model for prediction of recurrence (‘abnormal mitosis’ + ‘basal mitoses’ + ‘TNOM’ + ‘WHO grade’) achieved an AUROC of 0.7895 (*p* = 0.0003, 95% CI 0.6777 to 0.9013). Both these models outperformed conventional grading systems (Table 3).

## Discussion

This study highlights the potential importance of mitosis assessment and quantification in OED diagnosis and prognostication. Mitosis counting has been effectively implemented in the diagnosis of various malignancies [13, 17, 27–29], but its diagnostic importance in oral precancers remains largely unexplored. Due to the limitations of manual mitotic figure counting, PHH3 was explored to evaluate its role as a diagnostic and prognostic adjunct to conventional H&E assessment.

The role of various oncogenes in OED progression to cancer still remains unvalidated [30]. Ki-67 being a cell cycle marker, rather than a specific marker of mitosis, has shown conflicting results. In one study, the value of PHH3 and Ki-67 for measuring mitotic activity in OSCC demonstrated a significant association between expression of PHH3 (*p* = 0.016) and mitotic activity (*p* = 0.031) with survival time; however, no similar relationship was found with Ki-67 (*p* = 0.295) [31]. In another study, the presence, location and pattern of Ki-67 positivity demonstrated variable results for differentiation between normal tissue, OED and OSCC [32]. The unreliability of Ki-67 [32, 33] and the successful use of PHH3 as an independent biomarker in various different malignancies [13, 15, 17, 22, 34] led us to explore this marker further.

The TNOM was shown to increase proportionally with grade severity on both H&E and PHH3-IHC analyses, supporting findings in the existing literature [35–38. This could be explained by the increased stem cell turnover and quantity of abnormal mutations [39]. Overall, PHH3 mitotic count was greater than H&E, likely due to the inclusion of early prophase stage, which cannot be reliably distinguished on H&E-stained sections. In a previous study, a comparison in mitotic count between H&E and crystal violet-stained sections demonstrated significant differences between non-dysplastic oral mucosa, OED and OSCC [39]. Whilst our findings revealed a greater difference between mild and severe OED, control and high-grade/severe OED, promising differences were also observed between the more ‘demanding’ groups (moderate vs severe OED) in terms of mitosis number, mitosis type and mitosis location.

H&E analysis of mitosis type demonstrated a higher ratio of normal-to-abnormal mitoses in OED than control, which decreased with grade severity. Mitosis location assessment on H&E and IHC analysis demonstrated significant differences in the number of ‘basal’ and ‘suprabasal’ mitoses between grades. ‘Suprabasal’ mitoses were shown to be more predictive than ‘basal’ mitoses on PHH3-IHC. A study on meningioma demonstrated that PHH3 mitotic counts had a better interobserver correlation than H&E mitotic counts (*R*_m_ = 0.83 vs 0.77, respectively) [40], with good discrimination between grades (AUROC 0.91). Our study suggested similar findings, with better generally performance for PHH3-IHC models than H&E models, particularly for TNOM and mitosis location (Table 2). This is likely to be related to greater objectivity of mitosis assessment with PHH3 staining.

Prognostic models combining TNOM, mitosis type, location and histological grading showed better prediction for transformation and recurrence. Generally, the addition of clinical variables had minimal impact on model performances, whereas histological grading boosted predictive potential. Such a trend was also observed in a study by Mahmood et al. where inclusion of grades improved prognostic strength of histological OED models [10].

The most predictive H&E models for malignant transformation (‘abnormal mitoses’ + ‘suprabasal mitoses’ + ‘TNOM’ + ‘WHO grade’ = AUROC 0.8113) and OED recurrence (‘abnormal mitosis’ + ‘basal mitoses’ + ‘TNOM’ + ‘WHO grade’ = AUROC 0.7895) (AUROC 0.65) incorporated multiple mitotic features and outperformed conventional WHO grading on its own. In the case of PHH3-IHC models, the most superior models utilised fewer mitotic features for prediction of transformation (‘basal mitoses’ + ‘binary grading’ = AUROC 0.7714) and recurrence (‘TNOM’ + ‘WHO grading’ = AUROC 0.7783). These findings indicate that PHH3-IHC may be important for prognostication of OED, complementing H&E analysis.

The authors acknowledge a few limitations. First, the follow-up period comprised 5 years. Whilst transformation may occur later [41], a number of studies have shown transformation incidence to be highest during the first 5 years. [5, 41–44] A study by Hankinson et al. (2021) reported a median transformation time of 22 months (IQR 46.0) for a cohort of OED cases (*n* = 150) retrieved from the same centre as that used for this study [45]. Second, cases were from a single-centre, and the sample size could be regarded as small [46, 47]. However, the unit in question is a national tertiary centre providing service to a large geographical region, thereby increasing the biological diversity of the sample. Furthermore, the sample has an equitable distribution of dysplasia grades with inclusion of transformed and non-transformed cases. For an early exploratory study that serves as a basis for future work, our sample is similar to many other studies [31, 48, 49] of this kind. The control cases were included for clinical interest and early comparative analysis, hence the small numbers. They did not contribute to the prognostic work, which was the important and novel aspect of this study.

In conclusion, we report increased mitotic activity with OED progression. Mitotic quantification using PHH3-IHC is potentially more reliable than H&E analysis, with typically greater predictive strength, even with inclusion of fewer variables. The addition of histological grading further improved performance of PHH3-IHC models, more so than the H&E models. To the best of our knowledge, this is one of the first studies to utilise mitosis quantification and compare H&E with PHH3-IHC for OED analysis and prognosis prediction. The promising results call for further exploration of H&E and IHC markers to contribute to a more objective grading of OED and reliable prognosis prediction. Further studies with larger multicentre cohorts are required for clinical validation.

### Supplementary Information

Below is the link to the electronic supplementary material.

Supplementary Figure 1. ROC curves demonstrating the most predictive prognostic models (as highlighted inTable 2) for malignant transformation (A) and OED recurrence (B) based on H&E assessment (*n*=68, 340 ROI). AUROC= area under receiver operating characteristic. (PNG 174 kb)

High resolution image (TIF 9193 kb)

## Data Availability

All the data derived from this study are included in the manuscript. Further information for reasonable requests (if needed) can be made available, by contacting the last author (s.a.khurram@sheffield.ac.uk).

## Abbreviations

OED: Oral epithelial dysplasia
OSCC: Oral squamous cell carcinoma
HPV: Human papillomavirus
WHO: World Health Organization
H&E: Haematoxylin and eosin
PHH3: Phosphohistone-H3
IHC: Immunohistochemistry
WSI: Whole slide image
ROI: Region of interest
DPX: Dibutyl phthalate polystyrene xylene
ANOVA: Analysis of variance
AUROC: Area under receiver operator characteristic
TNOM: Total number of mitoses
Ki-67: Kiel-67
