# Supplementary figures and images for: Prognostic importance of mitosis quantification and PHH3 expression in oral epithelial dysplasia
Source: Virchows Arch. 2023 Oct 26;484(1):47–59. doi: 10.1007/s00428-023-03668-6 (PMC10791886; doi:10.1007/s00428-023-03668-6)

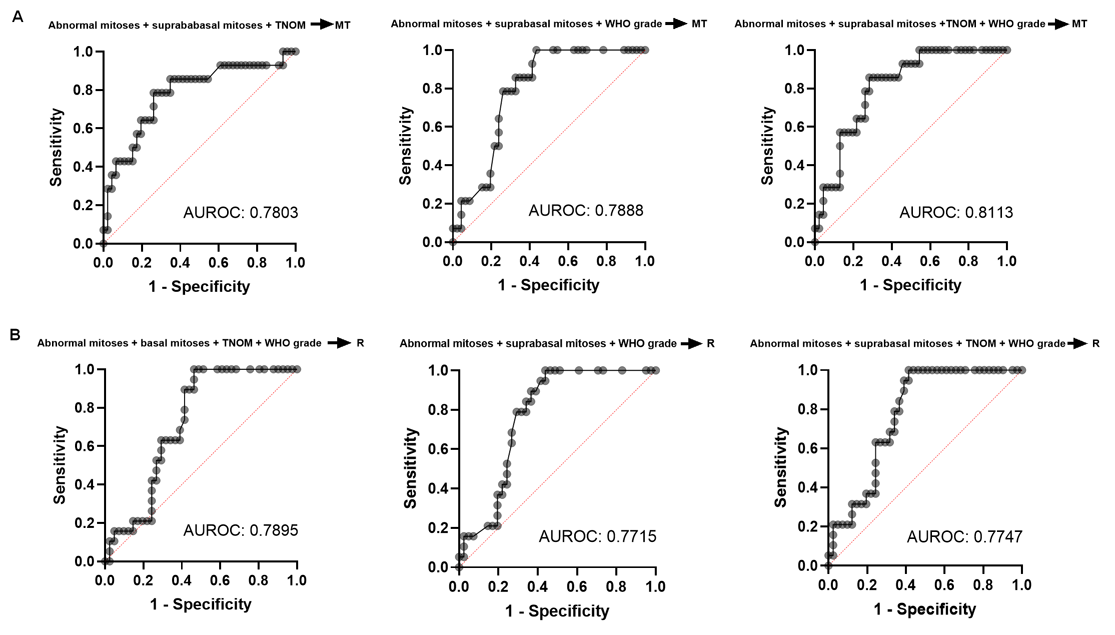

Supplement: Supplementary file 1 — Supplementary Figure 1. ROC curves demonstrating the most predictive prognostic models (as highlighted inTable 2) for malignant transformation (A) and OED recurrence (B) based on H&E assessment (n=68, 340 ROI). AUROC= area under receiver operating characteristic. (PNG 174 kb) [file 428_2023_3668_Fig4_ESM.png]

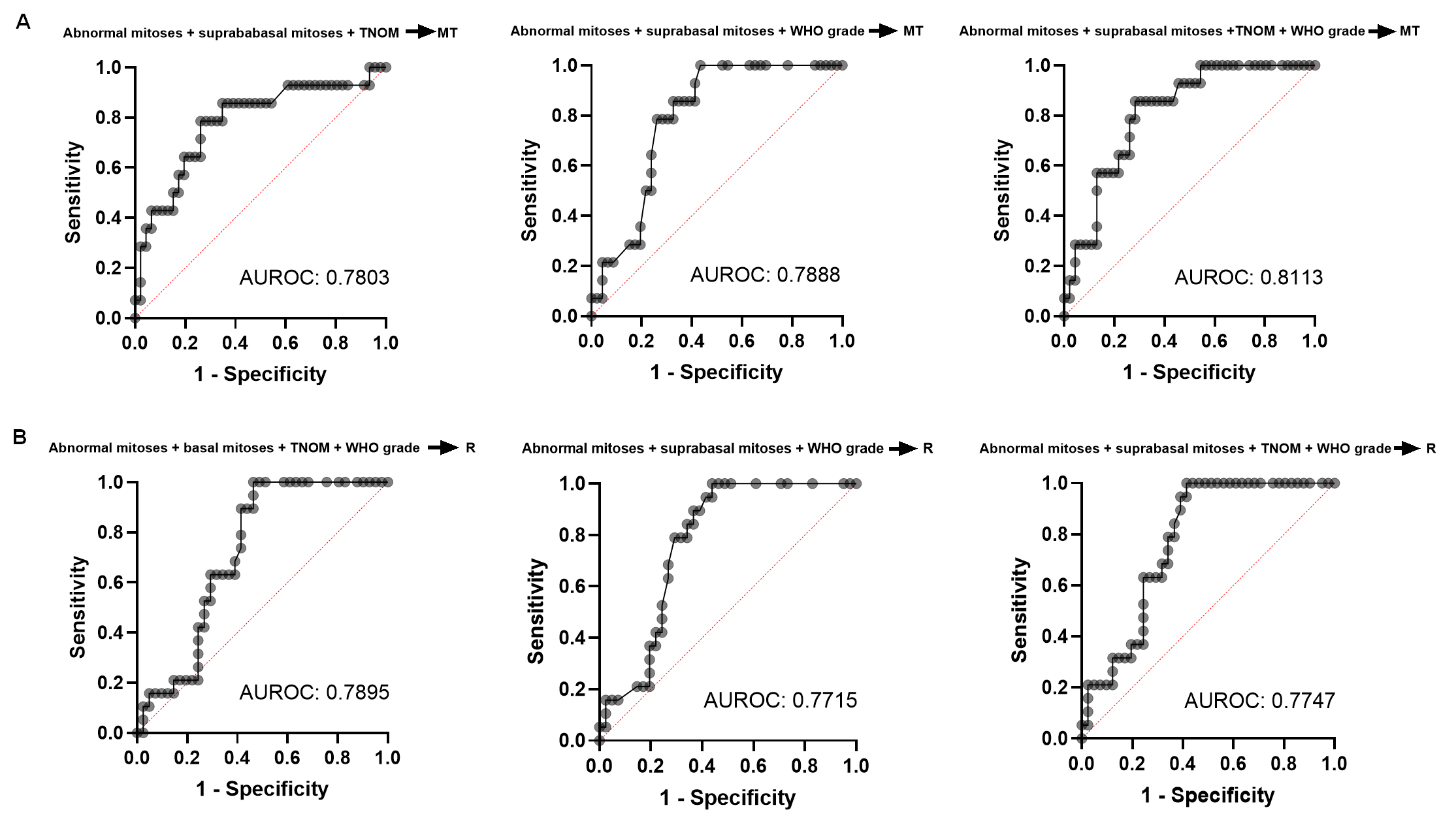

Supplement: Supplementary file 2 — High resolution image (TIF 9193 kb) [file 428_2023_3668_MOESM1_ESM.tif]
